# Ausgepreist: Zur Bedeutung von Proberäumen in der musikalischen Produktion und deren strukturellen Verknappung in Städten

**DOI:** 10.1007/s00548-023-00868-9

**Published:** 2023-05-23

**Authors:** Janet Merkel

**Affiliations:** Institut für Stadt- und Regionalplanung, Fachgebiet Stadt- und Regionalökonomie, TU Berlin, Hardenbergstr. 40 a, 10623 Berlin, Deutschland

**Keywords:** Arbeitsräume, Musikwirtschaft, Stadtentwicklung, Kulturraumschutz, Kulturpolitik, Rehearsal spaces, Music industry, Urban development, Cultural planning, Cultural policy

## Abstract

Proberäume als Räume der Musikwirtschaft erhalten bislang nur wenig Aufmerksamkeit, ob in der wirtschaftsgeografischen Forschung oder in infrastrukturellen Maßnahmen zur Unterstützung kultureller Produktion in Städten. Dabei erleben viele Städte eine strukturelle Verknappung von künstlerischen Arbeits- und Probenräumen durch Nutzungskonkurrenzen mit dem intensivierten Wohnungs- und Büroneubau in gegenwärtigen Stadtentwicklungsprozessen. Insbesondere die preiswerten lärmintensiven Nischen in ehemaligen Industrie- und Gewerbebauten verschwinden zunehmend. Der Artikel diskutiert Proberäume als kritische Infrastrukturen musikalischer Produktion und identifiziert verschiedene Aspekte für eine infrastrukturelle Förderung in den Städten. Der Grundlage des Artikels sind eine Literaturauswertung sowie sekundäranalytische Auswertungen aktueller Umfragen und Erhebungen zu Proberaumbedarfen unter Berliner Musikschaffenden von 2014 und 2021 durch die Senatsverwaltung für Kultur und Europa und das Musicboard sowie verschiedene Planungen und Beteiligungsverfahren zu künstlerischen Arbeitsräumen in Berlin. Dabei wird deutlich, dass die Verfügbarkeit, Bezahlbarkeit und die Angemessenheit von Proberäumen für Musikschaffende zu den zentralen Herausforderungen zählen und Städte bislang kaum Antworten auf die wachsende Raumkrise für künstlerische Arbeits- und Probenräume entwickelt haben.

## Abstract (en)

As spaces of the music economy, rehearsal spaces have received little attention in economic-geographical research or in infrastructural support for the production of culture in cities. However, many cities are experiencing a structural shortage of appropriate artistic work and rehearsal spaces owing to competition with intensified housing and office construction in current urban development processes. In particular, the low-cost noise emission-intensive niches in former industrial and commercial buildings are increasingly disappearing. The article discusses rehearsal spaces as critical infrastructures of musical production and identifies various aspects of infrastructural support in cities. The basis of the article is a literature review and secondary analysis of recent surveys and inquiries on rehearsal space needs among Berlin’s musicians from 2014 and 2021 by the Senate Department for Culture and Europe and the Musicboard, as well as various planning initiatives and participation processes for artistic workspaces in Berlin. It becomes clear that the availability, affordability, and appropriateness of rehearsal spaces for musicians are among the central challenges and that cities have hardly developed any answers to the growing space crisis for artistic work and rehearsal spaces.

## Einleitung

Die Auseinandersetzung mit den Räumen und der Räumlichkeit von Musik(wirtschaft) hat in der Wirtschaftsgeografie eine längere Tradition (Kloosterman 2005; Leyshon et al. 1998). Demnach benötigen Musiker:innen für den kreativen Schaffensprozess Interaktion und Austausch mit anderen Musikschaffenden, günstige Arbeits- und Probenräume sowie Präsentationsmöglichkeiten vor Publikum und Gatekeepern (Shaw 2013; Watson et al. 2009). Doch in vielen Städten verschwinden die Nischen preiswerter, lärmzulassender Räume. Der Beitrag bewegt sich an der Schnittstelle von Wirtschaftsgeografie und Stadtplanung und diskutiert am Beispiel Berlins die Situation von Proberäumen und zeigt darüber räumliche Steuerungsmöglichkeiten der Musikindustrie auf. Dabei wird *erstens* deutlich, dass öffentlich geförderte Infrastrukturen eine wichtige Rolle als Freiräume für die Entwicklung neuer Stile und Bands in der Musikwirtschaft und damit in der Nachwuchsförderung für kulturelle Innovation und Szeneproduktivität spielen. *Zweitens* sollten wirtschafts- und kulturpolitische Strategien die Produktionsseite der Musikwirtschaft stärker stützen. Und *drittens *ergibt sich daraus eine Notwendigkeit räumlicher Planungs- und Schutzstrategien in der Unterstützung der Musikindustrie (Dewey Muller 2020). Empirische Grundlage des Artikels sind eine Literaturauswertung sowie sekundäranalytische Auswertungen aktueller Erhebungen zu künstlerischen Arbeitsräumen in Berlin.

## Räume der Musikwirtschaft – Bedeutung und Potenzial von Proberäumen

Großstädte bilden die Zentren musikalischer Wertschöpfungsprozesse, sie sind zugleich Produktionsstandorte und Konsumentenmarkt. Die räumliche Dichte unterschiedlicher musikwirtschaftlicher Akteure und Institutionen fördert das Entstehen vielfältiger Produktionsnetzwerke und musikalischer Innovationen (Kuchar 2020). In der geografischen Forschung haben sich zwei Arten von Infrastrukturen als wichtige Rahmenbedingungen für die Musikproduktion herausgestellt: Einerseits die *materielle Produktionsinfrastruktur* von Proberäumen, kleinen unabhängigen Labels über Aufnahmestudios bis hin zu Live-Musikorten, an denen neue Songs und Auftritte vor Publikum erprobt werden können (Watson et al. 2009). Andererseits sind es *soziale Infrastrukturen,* die Begegnung, Austausch und den Aufbau von dichten kreativen (Produktions‑)Netzwerken ermöglichen und meist an informellen Orten wie Bars, Cafés oder Clubs entstehen (Currid 2007). Oft überlagern sie diese räumlich, wenn z. B. Live-Musikorte auch wichtige soziale Treffpunkte für musikalische Szenen sind (Whiting 2021). Als Teil der materiellen Produktionsinfrastruktur der Musikwirtschaft werden Proberäume in der geografischen Auseinandersetzung mit Räumen musikalischer Produktion und Wertschöpfung jedoch bislang vernachlässigt. Hier stehen Studios (Gibson 2005; Watson und Ward 2013), Clubs (Lange und Bürkner 2013) oder Live-Musikorte (Whiting 2021) im Zentrum wirtschaftsgeografischer Untersuchungen.

Proberäume nehmen wie Ateliers bildender Künstler:innen eine Schlüsselrolle in der künstlerisch-kulturellen Produktion als Orte situierter kreativer Praxis ein (vgl. Farías und Wilkie 2016). Für den musikalischen Schaffensprozess sind sie ein Ort der freien Entfaltung, des Experimentierens, des gemeinsamen Erprobens und des Entstehens musikalischer Neuheiten und Innovationen. Ob als umfunktionierte Garage oder Keller, Zimmer oder als angemietete Übungsräume, Proberäume sind zudem wichtig für informelle Lernprozesse, den Aufbau von Beziehungsnetzwerken und von performativem Kapital, also spezifischen Fertigkeiten und Wissen, um eine Bühnenpräsenz entwickeln zu können (Miller 2017). Als Arbeits- und Produktionsorte der „Kreativen“ (also der Urheber:innen und Künstler:innen) stehen sie am Anfang musikwirtschaftlicher Wertschöpfungsprozesse und umfassen den Bereich musikalischer „pre-production“ (vor der Aufnahme) als auch zunehmend der Produktion (DIW Econ 2020). Mit der Entwicklung von preiswerten digitalen Aufnahmetechniken, Softwarelösungen und Veröffentlichungsmöglichkeiten haben sich nicht nur die Geschäftsmodelle und Organisationsstrukturen der Musikwirtschaft verändert (Leyshon 2009; Reitsamer 2014; Tschmuck 2020), sondern Proberäume werden immer mehr zu Studios, in denen Bands ihre eigenen Songs produzieren und veröffentlichen können. Sie bilden somit in lokalen musikalischen Ökosystemen eine kritische Infrastruktur für das Entstehen neuer Musik (Homan 2014). Die Versorgung mit bezahlbaren Proberäumen stellt für Musikschaffende (ob Amateure, Semi-Professionelle oder Berufsmusiker:innen, mit fließenden Übergängen zwischen diesen verschiedenen Gruppen) eine wesentliche Rahmenbedingung für den Aufbau und die Entwicklung ihres künstlerischen Schaffens dar. Zugleich sind sie für den Erhalt lokaler musikalischer Ökosysteme und von Stadt als einem Standort künstlerischer Produktion relevant (Baker 2019; Hracs et al. 2011; Kuchar 2020). Wenngleich musikalische Arbeit durch neue technologische Entwicklungen und die Digitalisierung an sehr verschiedenen Orten stattfinden kann, bildet der Proberaum nach wie vor eine wichtige infrastrukturelle Basis für Musikschaffende. So erklärt Homan (2014, S. 154) zur Entwicklung des musikalischen Ökosystems Melbournes:

„For music practitioners, creative and cultural practice cannot be divorced from ‚liveability‘, and the survival of their favourite venues, retail stores, or recording and rehearsal studios. Strengthening these less glamorous connections is of equal importance to the current battles for planning principles on (for example) noise, replacing an obsessive league table culture with one that seriously addresses the quality of life for its musicians.“

Gegenwärtig erleben Städte eine wachsende Raumkrise, die Künstler:innen mit ihrem Bedarf nach Arbeits- und Proberäumen betrifft und immer häufiger zu Raumnutzungskonflikten führt (Schwegmann et al. 2021). Das *World Cities Culture Forum* erklärte den Erhalt und die Bereitstellung von Arbeits- und Proberäumen für die Kulturproduktion zum neuen kulturpolitischen Schwerpunktthema in Städten weltweit (BOP 2018). Die Coronapandemie hat diese Raumkrise zusätzlich verschärft und insbesondere die Live-Musik mit Clubs und kleinen Bühnen kritisch getroffen (Initiative Musik 2021). Als Ursache der Verknappung von Arbeits- und Proberäumen gelten gegenwärtige Stadtentwicklungsprozesse, die durch Wanderungsdynamiken und die zunehmende Finanzialisierung des Immobiliensektors vorangetrieben werden: die Aufwertung von Stadtquartieren, die bauliche Nachverdichtung in den Innenstädten und der Verlust von Industrie- und Gewerbegebieten zugunsten von Wohnnutzungen sowie der Verkauf von öffentlichen Liegenschaften, die preiswerte und lärmzulassende Nischen für Musikschaffende in den Städten verschwinden lassen (Der Regierende Bürgermeister von Berlin 2015; Pollio et al. 2021). Hinzukommt die gestiegene Nachfrage nach Gewerbeimmobilien, die auch bei Übungsräumen zu erheblichen Mietsteigerungen führt und musikalische Nutzungen verdrängt (Musicboard Berlin 2021). Während Metropolen wie Berlin mit dem „Arbeitsraumprogramm“ (seit 2016, vgl. Senatsverwaltung für Kultur und Europa 2022) oder London mit dem neuen „Cultural Infrastructure Planning“ (seit 2019, vgl. GLA 2019) die Versorgung mit Arbeitsräumen auf die kulturpolitische Agenda gesetzt haben, ist in kleineren Großstädten die Dringlichkeit oft noch nicht erkannt. Allein in Karlsruhe werden bis Ende 2025 mindestens 100 Proberäume für ca. 500 Musikschaffende durch Wohnungsneubau wegfallen (Hiegle 2022). Am Beispiel Berlins sollen im Folgenden aktuelle Entwicklungen verdeutlicht und Lösungsansätze kurz vorgestellt werden.

## Proberaumsituation für Musikschaffende am Beispiel Berlins

Berlin gilt als Impulsgeber für die deutsche Musikwirtschaft mit „10 renommierten Orchestern, zwei großen Konzerthäusern, vier Opern, vier Musical- und Revuetheatern, circa 1000 Orchestermusikern, 100 klassischen Ensembles und 880 Chören im E‑Musik-Bereich sowie 1000 Gruppen im Rock-Pop-Bereich, circa 1000 Jazzmusikern und 1200 DJs“ (Projekt Zukunft 2020, S. 6). Von den ca. 15.394 Erwerbstätigen in der Berliner Musikwirtschaft arbeiten mehr als 65 % freischaffend. In der Künstlersozialkasse waren 2021 9013 Personen als freie Musikschaffende gemeldet, davon 2369 als Popmusiker:innen (Musicboard Berlin 2021, S. 31). Die Versorgung mit Proberäumen bildet daher eine wichtige Produktionsbedingung für Musikschaffende in der Stadt.

Proberäume werden von verschiedenen Anbieter:innen bereitgestellt: Neben privaten Kleinvermieter:innen (z. B. in Kellern oder Gewerberäumen) und professionellen, meist hochpreisigen Proberaumanbieter:innen finden sich in Berlin viele gemeinnützige Initiativen wie das *ORWOhaus*, seit 2009 der größte selbstverwaltete Proberaumkomplex Europas mit 100 Proberäumen für mehr als 200 Bands (ORWOhaus 2022). Trotz dieser Vielfalt zeigen die Forschungen deutlich, dass öffentliche Infrastrukturen für Musikschaffende besonders wichtig sind: So untersuchen Hoyler und Mager (2005) die Rolle von soziokulturellen Zentren in Deutschland für die Entwicklung von Hip-Hop und zeigen, dass diese vor allem für Nachwuchsmusiker:innen wichtige Freiräume für die Entwicklung neuer Stile und Bands im Nachwuchsbereich bieten. Und für Musiker:innen im Amateurbereich kann eine Initiative wie „Lasst die Musik rein!“ des Landesmusikrates Berlin (2022) Kirchen, Stadtteilzentren, Bibliotheken oder Musikschulen für Proben öffnen.

Für die folgende Diskussion dienen zwei Studien zur Proberaumsituation professioneller Musikschaffender in Berlin als Grundlage einer sekundäranalytischen Auswertung: die *Ergebnisse der Umfrage zur Arbeitsraumsituation von Berliner Künstlerinnen und Künstlern* aus dem Jahr 2014 (Der Regierende Bürgermeister von Berlin 2015) und die *Berliner Proberaum Umfrage* des Musicboards Berlin von 2020 (Musicboard Berlin 2021). An der Arbeitsraumumfrage des Kultursenates nahmen 2207 Künstlerinnen und Künstler der Sparten Bildende Kunst, Darstellende Kunst, Tanz und Musik online an der Befragung teil. Sie zeigte erstmals den akuten Mangel an Ateliers, Projekt- und Spielorten, Studios und Übungsräumen in den innerstädtischen Bezirken auf. 70 % der teilnehmenden 382 Musiker:innen gaben an, dass sie keinen Proberaum haben, weil sie sich keinen leisten können (Der Regierende Bürgermeister von Berlin 2015, S. 2). Die Proberaumstudie des Musicboards (2021) gibt einen Überblick zur Proberaumsituation von 948 Musiker:innen aus dem Bereich der Popmusik. Ein Drittel der Befragten gab an, dass Musik ihre Haupteinnahmequelle sei (ebd., S. 4). Die restlichen zwei Drittel der Umfrage-Teilnehmer:innen bestreiten ihren Lebensunterhalt nicht hauptsächlich durch ihre musikalische Praxis, sondern haben Zweitjobs, die ihr musikalischen Schaffen stützen – dies betrifft „insbesondere Nachwuchsmusiker:innen oder experimentell arbeitende Musiker:innen“ (S. 4). Knapp die Hälfte der Musiker:innen nutzt den Proberaum auch als Studio und gibt an, dass sie auf der Suche nach geeigneteren Räumen ist (Musicboard Berlin 2021, S. 13). Beide Studien verdeutlichen die Proberaumknappheit in der Stadt durch anhaltende Mieterhöhung, Kündigung oder Umwandlung. Die Einkommenseinbußen und Planungsunsicherheiten während der COVID-19-Pandemie und das dadurch gestiegene Prekaritätsrisiko der Musiker:innen verschärfen die Situation zusätzlich (Betzler et al. 2021; Marquardt und Hübgen 2021). Für Musikschaffende ergeben sich drei zentrale Herausforderungen in der Versorgung mit Proberäumen: die Bezahlbarkeit, Verfügbarkeit und Angemessenheit.

### Bezahlbarkeit

Wissenschaftliche Studien zeigen immer wieder, dass die meisten Musiker:innen nur über geringe Einkommen verfügen (Betzler et al. 2021; Homan 2014) und deshalb auf bezahlbare Proberäume angewiesen sind (vgl. Der Regierende Bürgermeister von Berlin 2015). Um die Kosten gering zu halten, werden Proberäume von Musiker:innen miteinander geteilt, untervermietet oder nur stundenweise angemietet (Musicboard Berlin 2021, S. 15). In der Proberaumumfrage des Musicboards werden zwischen 5 und 15 € pro Stunde und eine Raummiete von maximal 200 € im Monat als leistbar benannt (ebd., S. 17). Da viele Proberaumanbieter in Berlin bereits 15 € pro Quadratmeter als Monatsmiete verlangen, ergeben sich Unterstützungsbedarfe.

### Verfügbarkeit

Mit der voranschreitenden Entwicklung und Umwidmung ehemals brachliegender Gewerbe- und Industriebauten gehen viele Proberäume verloren, die als Zwischennutzungen entstanden sind und damit bisherige Förderansätze infrage stellen. Denn viele Städte präferieren temporäre Anmietungen, statt die Entwicklung dauerhafter Infrastrukturen im öffentlichen oder gemeinnützigen Besitz zu unterstützen (Kulturraum 2022; Pollio et al. 2021; Scott 2022). Dies führt zu einer Abhängigkeit der künstlerischen Raumangebote an die Entwicklungen auf dem Immobilienmarkt und zwingt die Musiker:innen oft in suboptimale Räume wie z. B. Lagerhallen, feuchte Keller oder in Lagen außerhalb der Innenstadt. In Berlin konzentrieren sich die Proberaumanbieter zunehmend außerhalb des S‑Bahnrings (vgl. hierzu die Karte der Proberaumanbieter:innen in der Studie des Musicboards (2021, S. 5)). Bislang gibt es kaum langfristige Lösungsansätze, mit denen Proberäume dauerhaft gemeinnützig betrieben werden könnten (vgl. etwa gegenwärtige Entwicklungen in London mit der Einrichtung des Creative Land Trust (2020), der dauerhaft Studio- und Probenkomplexe in Gemeinnützigkeit ermöglicht).

### Angemessenheit

**Abb. 1:**
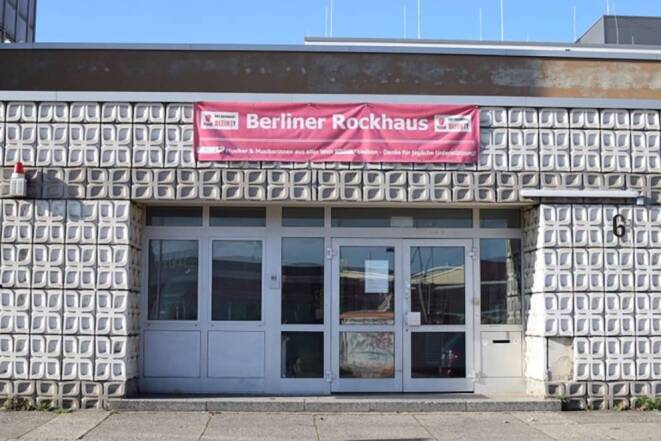
Eingang zum Berliner Rockhaus in Lichtenberg, 2022, *Foto*: Janet Merkel

Musikalische Probenräume müssen Lärm zulassen können. Dieses Merkmal unterscheidet sie von vielen anderen künstlerischen Arbeits- und Proberäumen (vgl. Bingham-Hall und Kaasa 2018). Zudem sollten sie heizbar und nicht feucht sein, um die elektronische Ausstattung und die Instrumente nicht anzugreifen. Knapp die Hälfte der 948 befragten Musiker:innen in der Proberaumumfrage ist aktuell auf der Suche nach geeigneteren Räumen. Als häufige Gründe werden „mangelhafte räumliche Gegebenheiten, insbesondere mangelhafte Infrastruktur, hygienische Zustände oder Instandhaltung, z. B. eine mangelnde Belüftung und Beheizung, ein fehlender Fahrstuhl und unzureichende, unhygienische Sanitäranlagen“ (Musicboard Berlin 2021, S. 15) angegeben. Zudem werden von den Musiker:innen ein „schlechter Schallschutz“ und „fehlende Aufbewahrungsmöglichkeiten“ genannt. Darüber hinaus bildet die „Anbindung an den öffentlichen Nahverkehr“ ein wichtiges Kriterium, da sich das Angebot zunehmend in die äußeren Stadtbezirke verschiebt, die in den Abend- und Nachtstunden mit ÖPNV erreichbar sein müssen. Viele wünschen sich zudem eine Rund-um-die-Uhr-Nutzung des Proberaums (ebd., S. 21). (Abb. 1 und 2).

**Abb. 2:**
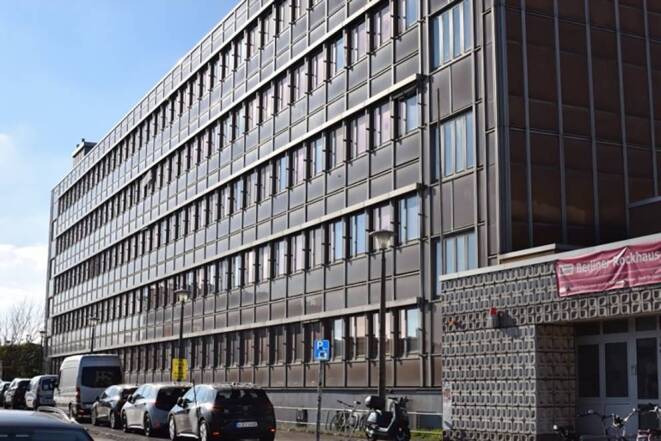
Berliner Rockhaus in Lichtenberg, 2022, *Foto*: Janet Merkel

## Räumliche Steuerungsmöglichkeiten in Städten

An der Diskussion der drei Kriterien Bezahlbarkeit, Verfügbarkeit und Angemessenheit wird deutlich, dass die Nachfrage nach preiswerten Proberäumen für professionelle Musiker:innen das verfügbare Angebot weit übersteigt und durch gegenwärtige Stadtentwicklungsprozesse weiter verknappt wird, sodass in Städten mehr unterstützende Maßnahmen benötigt werden, die sich im Dreieck von Kultur‑, Wirtschafts- und Stadtentwicklungspolitik bewegen könnten.

### Förderstrategien von Arbeits- und Proberäumen für musikalische Arbeit

Im Vergleich mit der klassischen Musik (und den Ausgaben in der Kulturpolitik für Orchester, Konzert- und Opernhäuser) genießt die populäre Musik bislang sehr viel weniger Aufmerksamkeit in der Kultur- und Wirtschaftspolitik, obwohl mit dem Diskurs um die Kreative Stadt und Kultur- und Kreativwirtschaft gerade die ökonomische Verwertbarkeit von Musik in den wirtschaftspolitischen Fokus gerückt ist (Barber-Kersovan et al. 2014). Berlin hat 2013 ein Musicboard als zentrale Anlaufstelle für Musikschaffende eingerichtet, das die Produktionsförderung von Bands, verschiedene Festivals und zunehmend auch die Vermittlung von geförderten als auch nichtgeförderten Proberäumen koordiniert (Lücke und Jóri 2019). Auch hat der Kultursenat bereits bei drohenden Proberaumverlusten eingegriffen. Nach Protesten der Musiker:innen konnte 2019 im Auftrag des Kultursenats treuhänderisch eine öffentliche Gesellschaft als Generalmieter für das *Rockhaus Berlin *eingesetzt werden, nachdem der Eigentümer dem bisherigen Betreiber gekündigt hatte, obwohl dieser bereits in umfangreiche schallschutz- und brandschutztechnische Baumaßnahmen investierte. Mit der treuhänderischen Übernahme können die 189 Räume für die nächsten 20 Jahre weiterhin an mehr als 1000 Musiker:innen vermietet und mit Mietkostenzuschüssen von 2,50 € pro Quadratmeter unterstützt werden. Allerdings wurde eine Staffelmiete mit Mietsteigerungen auf 21 € pro Quadratmeter vereinbart, die für viele der Bands nicht mehr leistbar ist (Morgenstern 2019). Neben dem schnellen Eingreifen im Falle von Proberaumverlusten durch Mieterhöhungen, Kündigungen oder Umwandlungen bedarf es zunehmend langfristiger Strategien, um die Proberaumbedarfe quantitativ und qualitativ abzudecken. Seit 2016 wird in der Senatsverwaltung für Kultur und Europa (2022) an einem übergreifenden Arbeitsraumprogramm für alle künstlerischen Sparten gearbeitet. Bislang gibt es hierüber aber nur 18 dauerhaft gesicherte Proberäume für Musiker:innen (Musicboard Berlin 2021, S. 23). Demgegenüber stehen allein über 2300 professionelle Popmusiker:innen. Mit Vertreter:innen der Koalition der Freien Szene wurde 2020 die gemeinnützige Kulturraum GmbH als Tochter der öffentlich-rechtlichen Stiftung für Kulturelle Weiterbildung und Kulturberatung gegründet, die nun die operative Verantwortung für die Entwicklung des Arbeitsraumprogramms trägt (Kulturraum 2022). Die Arbeit beschränkt sich im Moment auf die Akquise temporär anmietbarer Räume in etablierten Proberaumstandorten, die dann über das Arbeitsraumprogramm an professionell tätige Künstler:innen mit einer Förderung vergeben werden können. Zum aktuellen Zeitpunkt (Ende März) gibt es nur eine aktuelle Ausschreibung für ein Angebot in einem Tanzstudio.

### Planerische Steuerungs- und Schutzmöglichkeiten

Formelle und informelle planerische Instrumente spielen eine zentrale Rolle für räumliche Schutzstrategien in der Arbeits- und Proberaumversorgung. Das Kulturamt der Stadt Köln hat 2020 einen umfassenden Maßnahmenkatalog herausgegeben, der aufzeigt, wie sich mit planerischen Instrumenten kulturelle Nutzungen erhalten lassen: Diese reichen von Nutzungsvorgaben, veränderten Flächennutzungsplänen und Gebietskategorien, neuen Zuordnungen in der Baunutzungsverordnung bis zu neuen Regeln in städtebaulichen Verträgen, die neben sozialen auch kulturelle Nutzungen festschreiben könnten (Dewey Muller 2020). Zudem können Lärm- und Schallschutzinitiativen angeregt werden, etwa durch Fördermaßnahmen zum Umbau von Proberäumen und neue rechtliche Regeln für den Wohnungsneubau, wie durch das „Agent of Change“-Prinzip, das 2014 im australischen Planungsrecht eingeführt wurde (Roberts 2018). Darüber können bestehende lärmerzeugende Nutzungen wie Proberäume, Clubs und Live-Music-Orte in angrenzenden Neubauprojekten stärker berücksichtigt und vor einer anschließenden Verdrängung geschützt werden. Aus den Ergebnissen der zwei Berliner Studien wird zudem deutlich, dass neben räumlichen Schutzstrategien auch eine begleitende soziale Wohnungspolitik von hoher Relevanz ist, da die meisten Musiker:innen in der eigenen Wohnung proben und produzieren und hier ebenfalls auf bezahlbare Mieten angewiesen sind (Musicboard Berlin 2021; Reitsamer 2014).

## Fazit

Proberäume als Orte situierter kreativer Praxis sind kritische Infrastrukturen musikalischer Produktion, so das Argument dieses Beitrags. Sie übernehmen soziale, kulturelle und ökonomische Funktionen in musikalischen Wertschöpfungsprozessen und haben eine Bedeutung für die Stadtentwicklung, die Attraktivität von Standorten und als Wirtschaftsfaktor (Baker 2019). Bislang spielen sie aber nur eine untergeordnete Rolle in städtischen Kultur- und Wirtschaftsstrategien. Trotz der steigenden Knappheit von künstlerischen Arbeits- und Produktionsräumen in vielen Städten werden bislang kaum geeignete Förderinstrumente und Fördermaßnahmen entwickelt. Zu den gegenwärtigen Herausforderungen für Musikschaffende zählen sowohl die Verfügbarkeit, die Bezahlbarkeit als auch die Angemessenheit von Proberäumen. In der wirtschaftsgeografischen Forschung werden Proberäume als Räume der Musikwirtschaft kaum erforscht, insbesondere fehlen Analysen zu ihrer Rolle in kreativen Prozessen und lokalen musikalischen Ökosystemen. Zudem ist wenig untersucht, wie die Digitalisierung neue räumliche Unabhängigkeiten von musikalischen Zentren ermöglichen kann (Ballico 2021; Hracs et al. 2011; Watson 2020). Am Beispiel Berlins wurde kurz erläutert, welche konkreten Herausforderungen sich für Musikschaffende ergeben und welche Lösungsansätze und räumlichen Steuerungsmöglichkeiten bereits umgesetzt werden. Vor dem Hintergrund einer zunehmenden Verdrängung von musikalischen Räumen in den Städten bedarf es langfristig gesicherter öffentlicher oder gemeinnütziger Infrastrukturen, eines stärkeren Fokus auf die Produktionsbedingungen in der Musikwirtschaft und der Anwendung planerischer Instrumente, um Arbeits- und Produktionsräume als wesentlichen Bestandteil der kulturellen Infrastruktur in Städten zu sichern und zu schützen.
